# The Brain-Derived Neurotrophic Factor Val66Met Polymorphism Moderates the Effects of Childhood Abuse on Severity of Depressive Symptoms in a Time-Dependent Manner

**DOI:** 10.3389/fpsyt.2016.00151

**Published:** 2016-08-29

**Authors:** Caitlin Webb, Jane M. Gunn, Maria Potiriadis, Ian P. Everall, Chad A. Bousman

**Affiliations:** ^1^Department of Psychiatry, The University of Melbourne, Parkville, VIC, Australia; ^2^Department of General Practice, The University of Melbourne, Parkville, VIC, Australia; ^3^NorthWestern Mental Health, Melbourne, VIC, Australia; ^4^Florey Institute of Neuroscience and Mental Health, The University of Melbourne, Parkville, VIC, Australia; ^5^Centre for Human Psychopharmacology, Swinburne University of Technology, Hawthorne, VIC, Australia

**Keywords:** depression, gene–environment, brain-derived neurotrophic factor, childhood adversity, stressful life events, longitudinal cohort

## Abstract

Cross-sectional studies have demonstrated that the brain-derived neurotrophic factor (*BDNF*) Val66Met single-nucleotide polymorphism moderates the association between exposure to negative life events and depression outcomes. Yet, it is currently unclear whether this moderating effect is applicable to positive life events and if the moderating effect is stable over time. To address these gaps in the literature, we examined clinical and *BDNF* genotypic data from a 5-year prospective cohort of 310 primary care attendees. Primary care attendees were selected based on existence of depressive symptoms at screening. Depressive symptoms were assessed at baseline and annually for 5 years post-baseline using the Primary Care Evaluation of Mental Disorders Patient Health Questionnaire-9 (PHQ-9). Linear mixed models assessed differences in depressive symptom severity over the 5-year follow-up period by *BDNF* Val66Met and history of life events, both negative and positive. Analysis identified a novel three-way interaction between the *BDNF* Val66Met polymorphism, history of severe childhood abuse, and time. *Post hoc* analysis stratified by time showed a two-way interaction between Val66Met and severe childhood abuse at baseline that was not detectable at any other time point. An interaction between Val66Met and positive life events was not detected. Our longitudinal results suggest that the *BDNF* Val66Met polymorphism moderates the depressive symptom severity experienced by those with a history of severe childhood abuse but does so in a time-dependent manner. Our results further support the notion that gene–environment–depression interactions are dynamic and highlight the importance of longitudinal assessment of these interactions. Given these novel longitudinal findings; replication is required.

## Introduction

The etiologies of depression are poorly understood, with the most recent etiological theories focusing on gene–environment interactions. Depressive phenotypes have long been associated with exposure to negative events, yet phenotypic variation is evident, with individuals exposed to the same environmental stress either not developing depressive symptoms or developing a range of symptom severity (1). This phenotypic variation is the basis of the diathesis–stress psychological framework (2), which suggests individuals possess “risk” factors (e.g., genetic variants), which make them more vulnerable to developing psychological symptoms when exposed to an adverse event. Historically, the diathesis–stress framework has guided research into gene–environment interactions. However, an emerging alternative framework is that of differential susceptibility. The differential susceptibility framework postulates that traditionally defined “risk” factors may better be defined as “phenotypic plasticity” factors in that individuals carrying these factors are more sensitive to both positive and negative environmental influences (3).

Environmental influences consistently linked to depression include adverse childhood events (4, 5) and current domestic abuse among women (6). However, environmental stress alone has been identified as insufficient to cause depression (7), emphasizing the gene–environment hypothesis. Current literature clearly identifies numerous genetic variants associated with mediating the differential response to negative life events in the development of depression (8). Among these genes is the brain-derived neurotrophic factor (*BDNF*), specifically the Met allele in the Val66Met single-nucleotide polymorphism located in the coding region of exon 2 (rs6265 – A66G) (8). *BDNF* is a pro-survival factor, involved in brain cell survival and proliferation (8) and has also been suggested to affect neuronal plasticity (9). Depressed individuals are shown to have decreased serum BDNF levels compared to controls (10–12) and carriers of the *BDNF* rs6265 Met allele show a reduction in *BDNF* activity (13).

Studies examining the interaction between *BDNF* genetic variation and stressful life events have reported conflicting results. A number of studies identified an association between the *BDNF* Met allele and greater depressive symptom severity in the context of childhood abuse or adult abuse (14). Other studies reported that *BDNF* moderates the response to some, but not all, forms of abuse (15, 16). While two large studies, one a cross-sectional study (17) and another a case–control study (18) reported no moderating effect of *BDNF* genetic variation on the association between stressful life events and depressive symptoms.

In light of these conflicting results, the role of *BDNF* genetic variation in moderating depressive symptoms in the context of stressful life events remains unclear. In addition, the role *BDNF* plays in moderating depressive symptom severity in the context of life events over time is not clear. As such, we examined whether the *BDNF* Val66Met polymorphism moderated the relationship between life events (negative and positive) and depressive symptom severity in a 5-year prospective cohort of 310 primary care attendees.

## Materials and Methods

### Participants

Participants were recruited from the Diagnosis, Management and Outcomes of Depression in Primary Care (*diamond*) study, an ongoing prospective cohort that commenced in 2005 (19). The *diamond* study aims to document the experiences, health outcomes, treatment, and service usage of primary care attendees identified as having clinically relevant depressed mood at screening; with patients recruited from 30 rural and metropolitan general practices randomly recruited in Victoria, Australia (19). Primary care patients were eligible for the *diamond* cohort if they were: (a) aged 18–75 years, (b) able to read English, (c) not terminally ill, (d) did not reside in a nursing home, and (e) scored 16 or higher on the Center for Epidemiologic Studies Depression Scale (CES-D). Participants were assessed annually using postal surveys as well as computer-assisted telephone interviews. In 2011 (cohort year 6), participants enrolled in the cohort were invited to provide a saliva sample for DNA extraction and genotyping. All procedures were conducted in accord with principles expressed in the Declaration of Helsinki and obtained approval from the University of Melbourne Human Research Ethics Committee (Ethics ID 1135247.1).

### Depressive Symptom Measures

Longitudinal depressive symptoms were assessed at baseline and annually for 5 years thereafter using the self-administered Primary Care Evaluation of Mental Disorders Patient Health Questionnaire-9 (PHQ-9) (20). The PHQ-9 is based directly on the nine signs and symptoms of major depressive disorder as described in the Diagnostic and Statistical Manual of Mental Disorders-IV (DSM-IV) (21) and has been validated to screen and monitor depression severity in the primary care setting (22). The PHQ-9 asks respondents to rate their symptoms over the past 2 weeks and is scored on a scale of zero (“not at all”) to three (“nearly every day”) for each item with a range of 0–27 (20). Scores of 5, 10, 15, and 20 on the PHQ-9 represent cut-points for mild, moderate, moderately severe, and severe depression, respectively (20). DSM-IV criteria (23) for major depressive disorder was assessed using the Composite International Diagnostic Interview (CIDI) Auto version 2.1 (24) by a trained research assistant.

### Childhood Abuse

At baseline exposure to physical and sexual childhood abuse was measured using the Child Maltreatment History Self-Report (CMHSR) (25). The CMHSR consists of 11 items, 7 pertaining to physical abuse and 4 to sexual abuse. The physical abuse questions have a four-point response option (never, rarely, sometimes, often), whereas the sexual abuse questions have a yes/no response option. Severe abuse was defined using the scoring algorithm previously described by Macmillan and associates (25).

### Partner Abuse

History of partner abuse in adulthood was measured using a history of partner fear as a proxy marker. This was done because the available composite abuse scale (CAS) in our cohort has not been validated in males as well as to minimize the risk of under-reporting associated with the CAS and to reflect the importance on an individual’s perception in the experience of abuse (26).

### Life Events

At baseline, participants completed a 13-item life events questionnaire adapted from the Life Experiences Scale and Life Events Questionnaire (27, 28). A full list of events measured is displayed in Table S1 in Supplementary Material. For each life event, participants were asked to indicate whether or not they experienced the life event in the past 12 months and if so, what impact the event had on them. There were six possible responses for each life event: (1) No, I have not experienced this event in the past 12 months; (2) Yes, and it has had an extremely negative impact on me; (3) Yes and it has had a slightly negative impact on me; (4) Yes but it has had no impact on me; (5) Yes and it has had a slightly positive impact on me; (6) Yes and it has had an extremely positive impact on me. In this study, presence of a positive event was defined as one or more events reported as slightly or extremely positive. Presence of a negative event was defined as one or more events reported as slightly or extremely negative.

### Potential Confounding Variables

At baseline, potential confounding factors, including demographics, smoking status, quality of life (29), and self-rated health status (30) were assessed. Panic and other anxiety syndromes were also assessed using the anxiety module of the PHQ (22). Alcohol and drug abuse/dependence (i.e., cannabis, opioid, sedative, cocaine, amphetamine, hallucinogens, inhalants) was assessed using the CIDI Auto version 2.1 (24). At each assessment point, the use of antidepressants, anxiolytics, and antipsychotics, as well as self-reported visits in the past 12 months to a psychiatrist and/or psychologist were also assessed.

### DNA Extraction and Genotyping

DNA extraction and genotyping details have previously been published (31). In brief, DNA was recovered from stabilized saliva samples and the rs6265 polymorphism was genotyped as part of a larger genotyping project with the Sequenom MassARRAY MALDI-TOF genotyping system (Sequenom Inc., San Diego, CA, USA). To detect for the presence of population stratification, 60 unlinked ancestry-informative markers (AIMs; Table S2 in Supplementary Material) representing the three HapMap phase III populations (Northern/Western European, Han Chinese, and Yoruba in Nigeria) were also genotyped (32).

### Statistical Analysis

Chi-square analysis of *BDNF* genotype was used to detect departures from Hardy–Weinberg equilibrium. Due to the low number of Met/Met carriers (*n* = 9), individuals who carried the Met allele (Val/Met or Met/Met) were compared to homozygous Val carriers. To estimate the presence of population stratification, the 60 AIMs were used to assign each participant to the HapMap ancestral group (Northern/Western European, Han Chinese, and Yoruba in Nigeria) for which they carried the greatest proportion of that population’s AIMs.

Linear mixed models were used to determine trajectory differences in PHQ-9 depressive symptom severity over the 5-year follow-up period by genotype and abuse/life event type. The mixed models approach enables use of all repeated measurements, accounts for clustering of participants within primary care sites, and provides unbiased estimates in the presence of missing data. Prior to creation of interaction terms and modeling, genotypes, abuse, and life event variables as well as covariates were centered (33). Potential covariates were assessed for their association with each of the abuse/life event types and genotypes using chi-square, Fisher’s exact, or analysis of variance tests, depending on the variable structure. Covariates with *p*-values ≤0.05 were retained for adjusted analysis (Table S3 in Supplementary Material).

The unadjusted model included fixed effects of time, genotype, abuse/event, and a time × genotype, time × abuse/event, genotype × abuse/event, and time × genotype × abuse/event interaction terms. Random effects included individual, primary care site, intercept (PHQ baseline score), and slope (time). Unadjusted models that returned significant main effects (*p* < 0.05) were then adjusted for covariates to account for any bias from baseline characteristics. Adjusted models included relevant covariates as well as covariate × time, covariate × genotype, and covariate × abuse/event type interaction terms as previously recommended (34). Covariance models used for the random and repeated effects were unstructured and first-order autoregressive, respectively.

Adjusted models were then refined using a backward stepwise penalized likelihood model selection strategy by starting with the most complex model and removing the term with the largest *p*-value above 0.05 using unrestricted maximum likelihood estimations. If removal of a covariate term increased the Bayesian information criterion (BIC) compared to previous more complex model the covariate was retained. Missing data of longitudinal measurements were assumed missing at random. All analyses were performed using SPSS 21.0 (IBM, Armonk, NY, USA).

## Results

### Sample Characteristics

**Figure 1:**
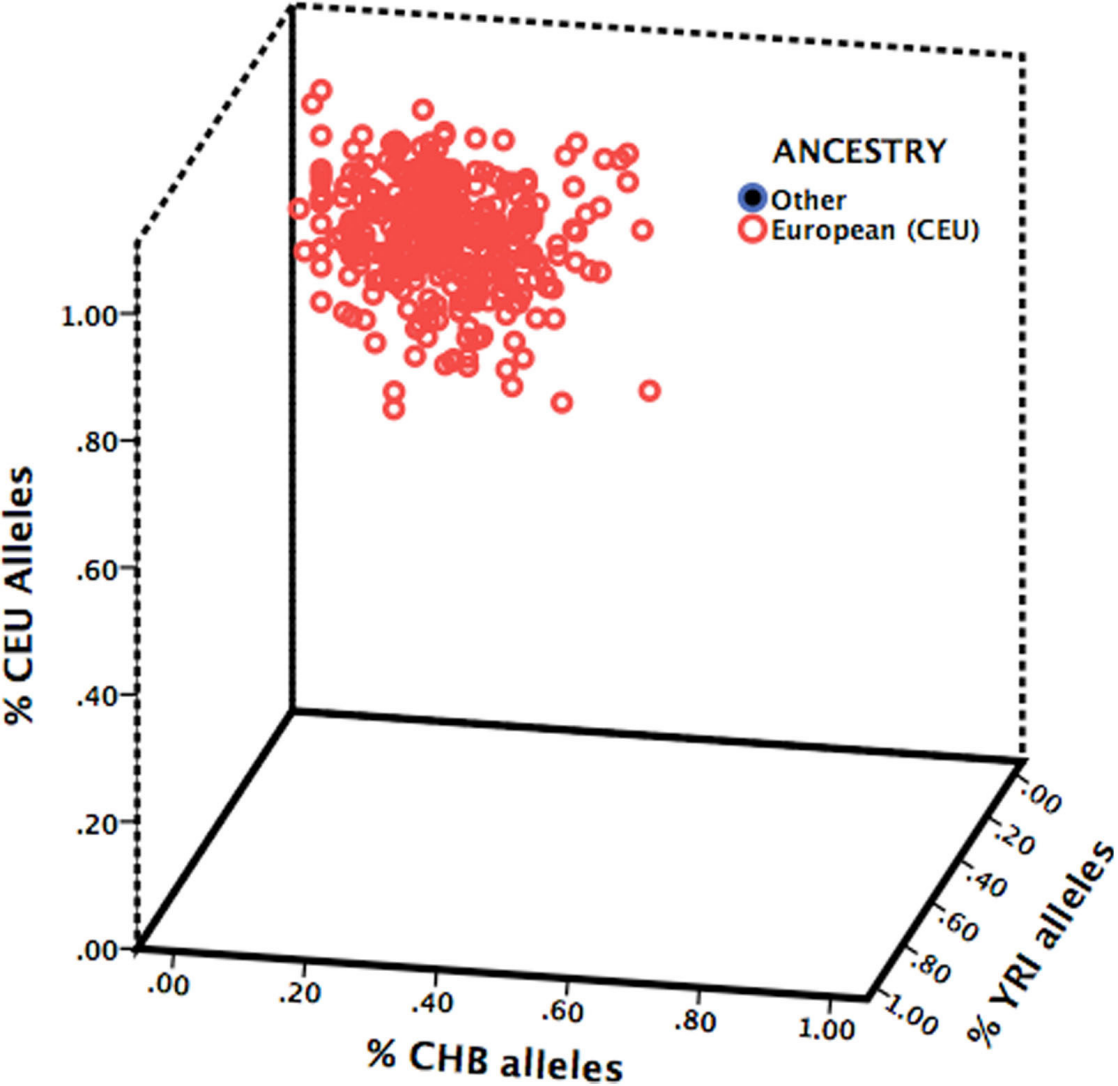
**Ancestry estimation of study sample**. Participants were assigned to the HapMap ancestral group (Northern/Western European, CEU; Han Chinese, CHB; or Yoruba in Nigeria, YRI) for which they carried the greatest proportion of that population’s ancestry-informative markers.

A total of 789 participants were recruited into the diamond cohort of whom 498 were enrolled at the time of DNA collection (cohort year 6) and 344 (69%) consented and returned a DNA sample. Individuals missing genotype data (*n* = 22) or abuse data (*n* = 13) were excluded. A total sample of 310 participants was included in the analysis (Table 1). Genotype frequencies for *BDNF* rs6265 were 67% Val/Val, 33% Met carriers (30% Val/Met and 3% Met/Met) and were in Hardy–Weinberg equilibrium (*p* = 0.768). All participants were of Northern/Western European (CEU) ancestry based on 60 unlinked AIMs (Figure 1).

**Table 1:** **Participant characteristics (*n* = 310)**.

Baseline variables	
*Demographics*	
Age, mean (SD) years	48.9 (11.9)
Sex, % (*n*) female	71 (220)
English as first language, % (*n*)	99 (307)
Northern European genetic ancestry, % (*n*)	100 (310)
*Depression history*	
DSM-IV major depressive disorder, % (*n*)^a^	49.2 (149)
Relative with a history of depression, % (*n*)^b^	75.4 (193)
*Co-morbid substance use*	
Smoker, % (*n*)^c^	23.9 (74)
Alcohol abuse/dependence, % (*n*)^a^	13 (39)
Substance abuse/dependence (excludes alcohol), % (*n*) – 301	6.3 (19)
*Medication use*	
Antidepressant, % (*n*)	40.6 (126)
Anxiolytic, % (*n*)	9.7 (30)
Antipsychotic, % (*n*)	5.5 (17)
Sedative, % (*n*)^c^	31.1 (96)
St John’s Wort, % (*n*)^c^	9.4 (29)
Visited counselor/psychologist/psychiatrist past 12 months, % (*n*)	33.2 (103)
Self-rated health, % (*n*) good to excellent	63.5 (197)
*Education*, % *(*n*)*^c^	
Completed year 12 or less	47.3 (147)
Diploma or certificate	25.2 (78)
Bachelor degree or higher	27.2 (84)
*Managing on available income*, % *(*n*)*^d^	
Easily/not too bad	48.1 (148)
Difficult some of the time	35.7 (110)
Difficult all of the time/impossible	16.2 (50)
*WHO Quality of Life and Functioning*	
Environmental context, mean (SD)	64.2 (13.1)
Social context, mean (SD)	49.7 (23.8)
*Severe childhood abuse*, % (*n*)	
Sexual	12.9 (40)
Physical	12.9 (40)
Either or both – sexual and/or physical	39 (121)
History of partner fear, % (*n*)	33.2 (103)
*Life events in past 12 months, %* (*n*)	
At least one positive	25.8 (80)
At least one negative	81.9 (254)

*^a^n = 301*.

*^b^n = 256*.

*^c^n = 309*.

*^d^n = 308*.

*WHO, World Health Organization*.

### Unadjusted Findings

Unadjusted linear mixed models for the *BDNF* rs6265 genotype across the abuse and life event types are summarized in Table 2. A two-way interaction was not found between *BDNF* rs6265 and positive events (*F*_1, 307_ = 0.12, *p*_unadjusted_ = 0.728) or *BDNF* rs6265 and negative events (*F*_1, 307_ = 0.04, *p*_unadjusted_ = 0.844). However, a two-way interaction was detected between *BDNF* rs6265 and severe childhood abuse (*F*_1, 307_ = 4.6, *p*_unadjusted_ = 0.031) and a three-way interaction was observed between the *BDNF* rs6265 genotype, severe childhood abuse, and time (*F*_1, 299_ = 5.4, *p*_unadjusted_ = 0.021).

**Table 2:** **General linear mixed model parameter *p*-values for main and interaction effects of BDNF rs6265 genotype, abusive experiences, and life events on 60-month depressive symptom trajectories (*n* = 310)**.

Unadjusted *p*-values for Type III fixed effects*							

Event type in model	BDNF rs6265	Event type	Time	rs6265 × event type	rs6265 × time	Event type × time	rs6265 × event type × time
History of severe child abuse	0.757	**0.001**	**0.001**	**0.031**	0.084	0.482	**0.021**
History of partner fear	0.777	**0.001**	**0.001**	0.090	0.332	0.104	0.636
*Life events*							
Positive	0.899	0.147	**0.001**	0.728	0.541	**0.026**	0.473
Negative	0.920	**0.008**	**0.001**	0.844	0.316	0.395	0.894

*Bolded values indicate p < 0.05*.

### Covariate-Adjusted Findings

**Figure 2:**
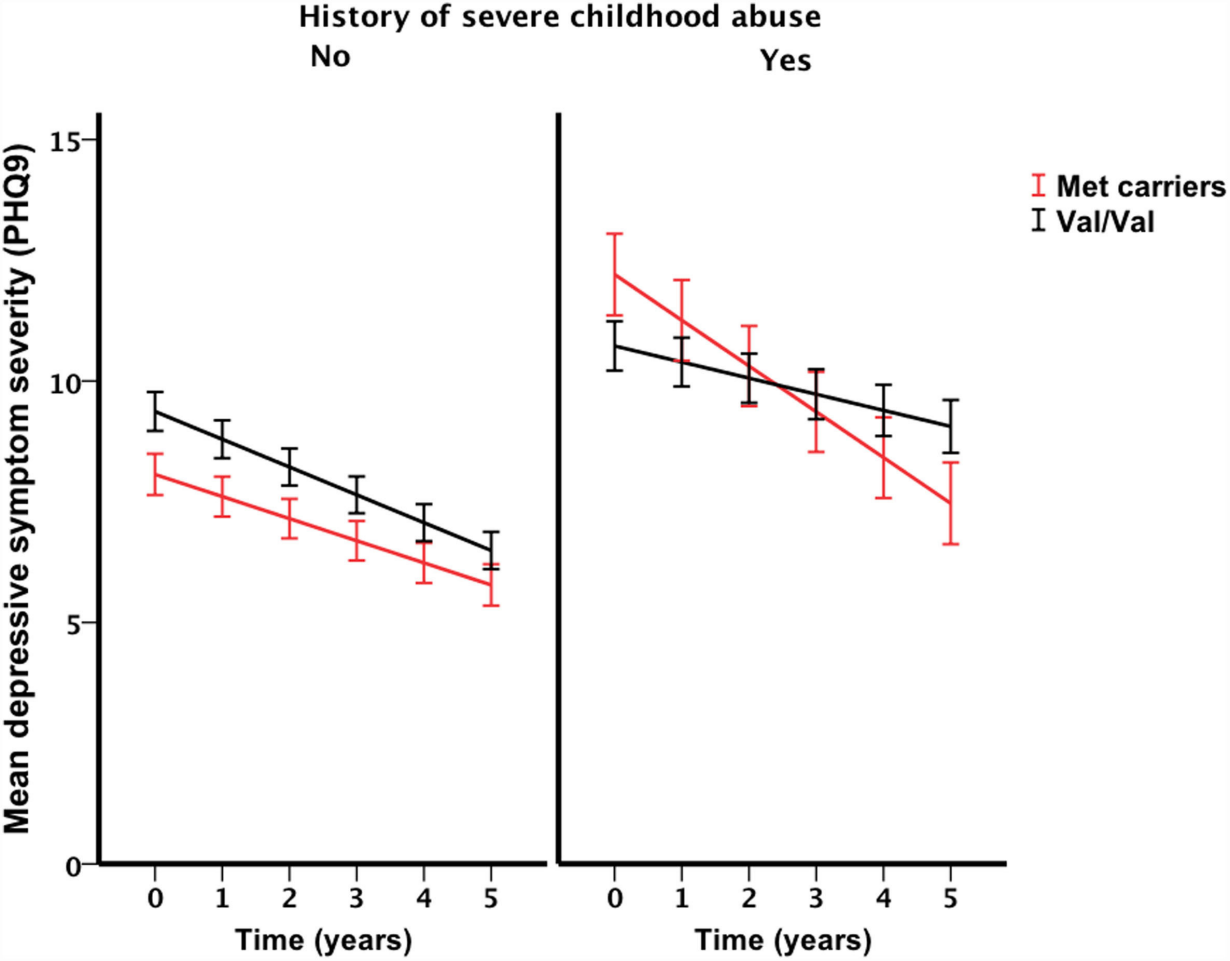
**Interaction effect of severe childhood abuse and *BDNF* Val66Met genotype on 5-year depressive symptom trajectories**. Baseline represented by time point 0, annual assessment thereafter. Points and associated SE bars represent predicted values based on the final covariate-adjusted model. PHQ-9 = Primary Care Evaluation of Mental Disorders Patient Health Questionnaire-9.

**Figure 3:**
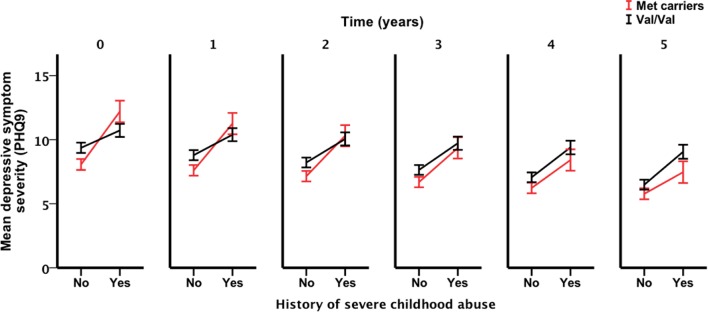
**Interaction effect of severe child abuse and *BDNF* rs6265 genotype by time point**. Time point zero represents baseline, measured annually from baseline. Points and associated SE bars represent predicted values based on the final covariate-adjusted model. PHQ-9 = Primary Care Evaluation of Mental Disorders Patient Health Questionnaire-9.

**Figure 4:**
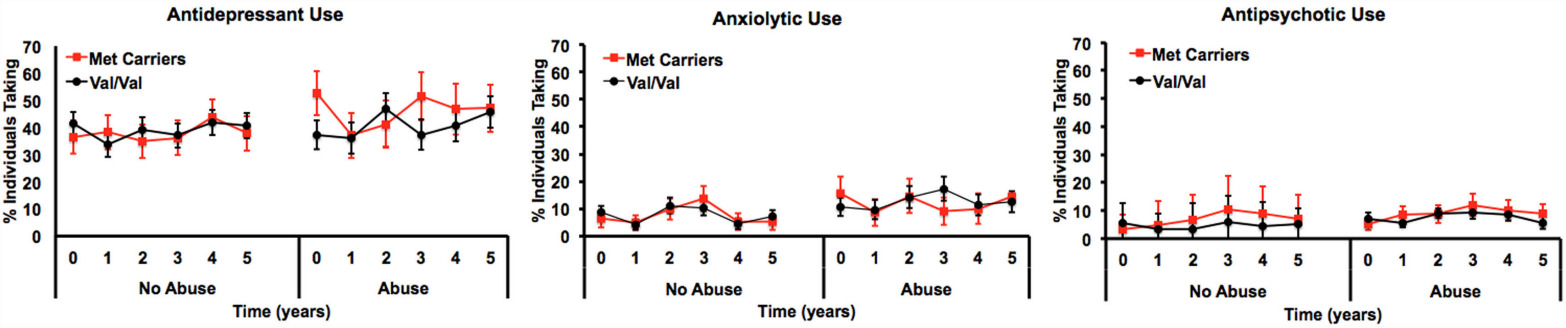
**Longitudinal measurements of medication usage by percent of individuals reportedly taking the medication by genotype and history of severe child abuse**.

**Figure 5:**
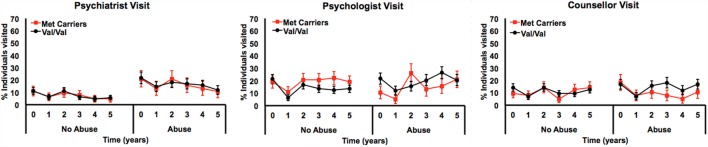
**Longitudinal measurements of psychosocial treatment by percent of individuals reported as having seen a psychiatrist, psychologist, or counselor once or more in the preceding 12 months, by genotype and history of severe child abuse**.

Only the three-way interaction survived adjustment for covariates (*F*_1, 292_ = 4.9, *p*_covariate adjusted_ = 0.027) (Table 3; Table S4 in Supplementary Material). Figure 2 shows Met allele carriers with a history of severe childhood abuse had a significantly greater reduction in depressive symptom severity over the 5-year follow-up period compared to their Val/Val carrying counterparts. *Post hoc* analysis stratified by time showed the two-way interaction between *BDNF* rs6265 and severe childhood abuse was present at baseline (*F*_1, 297_ = 5.4, *p*_unadjusted_ = 0.021) but no other time points (Figure 3). Importantly, further *post hoc* analyses suggested this time-dependent effect was not due to differential receipt of psychosocial (i.e., visits to counselor, psychologist, or psychiatrist) or pharmacological (i.e., antidepressant, anxiolytic, or antipsychotic) therapy over the 5-year study period (Figures 4 and 5).

**Table 3:** **Type III fixed effects^a^ for terms included in the final covariate-adjusted linear mixed model**.

Parameter	Degrees of Freedom		*F*	*p*
	Numerator	Denominator		
Intercept	1	310.128	356.731	**0.000**
Time	1	291.276	16.695	**0.000**
Genotype – rs6265	1	333.991	0.804	0.371
Severe child abuse	1	332.223	1.897	0.169
rs6265 × time	1	291.919	1.753	0.187
Severe child abuse × time	1	292.359	0.018	0.895
rs6265 × severe child abuse	1	305.182	2.786	0.096
rs6265 × severe child abuse × Time	1	292.215	4.925	**0.027**
DSM-IV Depression diagnosis	1	317.757	33.355	**0.000**
DSM-IV Depression diagnosis × Time	1	290.903	5.853	**0.016**
DSM-IV Depression diagnosis × rs6265	1	297.564	0.032	0.858
DSM-IV Depression diagnosis × severe child abuse	1	297.116	0.205	0.651
Self-rated health	1	307.656	38.684	**0.000**
Self-rated health × time	1	291.349	5.061	**0.025**
Self-rated health × rs6265	1	296.148	0.334	0.564
Self-rated health × severe child abuse	1	296.054	0.136	0.712
DSM IV substance abuse/addiction	1	306.156	4.014	**0.046**
DSM IV substance abuse/addiction × time	1	289.680	0.041	0.839
DSM IV substance abuse/addiction × rs6265	1	294.717	3.176	0.076
DSM IV substance abuse/addiction × severe child abuse	1	294.647	0.470	0.493
WHOQOL Social	1	300.581	18.361	**0.000**
WHOQOL Social × rs6265	1	299.536	3.026	0.083
WHOQOL Social × severe child abuse	1	297.433	1.061	0.304

*^a^Dependent variable: PHQ depressive symptom severity*.

*Bolded values indicate p < 0.05*.

## Discussion

We demonstrated a novel three-way interaction between the *BDNF* Val66Met polymorphism, a history of severe childhood abuse and time. This interaction withstood adjustment for key covariates and could not be attributed to differential receipt of psychosocial or pharmacological therapy. Previous studies have showed the *BDNF* Val66Met polymorphism moderates the relationship between history of childhood adversity and depression outcomes (15, 35, 36). Our longitudinal results not only support these previous findings but also suggest the moderating effect conferred by the *BDNF* Val66Met polymorphism may not be stable over time. In fact, we showed the presence of the interaction between *BDNF* rs6265 and severe childhood abuse was dependent on the time at which it was assessed; suggesting time as a key factor when testing gene–environment interactions.

The stability of gene–environment–depression interactions over time has only recently been described (37) but to our knowledge the current study is the first to demonstrate that these interactions may not be stable over time. Demonstrating the stability of a gene–environment interaction is critical to our conceptual and clinical understanding of depression and related outcomes. For example, at baseline, our analysis detected an interaction that is consistent with the differential susceptibility framework (3) in that Met allele carriers reported fewer depressive symptoms in the absence of severe childhood abuse and greater depressive symptoms in the presence of a history of severe childhood abuse, compared to Val/Val individuals who had similar depressive symptom severity regardless of their history of childhood abuse. However, over the course of 5 years, this interaction was gradually attenuated and support for the differential susceptibility framework was diminished. Furthermore, the presence of a time-dependent interaction may in part explain the sub-optimal reproducibility of *BDNF* by environment interactions in previous cross-sectional studies. Although other explanations for this interaction effect instability likely exist, we ruled out many of these explanations (e.g., therapy exposure) in our analyses. Nevertheless, further longitudinal examinations of *BDNF* by environment interactions are required to validate our findings.

### Potential Mechanism(s)

The mechanism(s) by which the *BDNF* rs6265 polymorphism moderates the relationship between severe childhood abuse and depressive symptom severity in adulthood is unclear and is beyond the scope of this study. However, previous studies have shown *BDNF* gene expression and serum protein levels are decreased in depressed individuals compared to healthy controls (10–12) and are also decreased in individuals with the Met allele (13), albeit in healthy individuals Val/Val carriers had decreased BDNF levels (38). Nevertheless, there is modest evidence for a link between peripheral *BDNF* levels and depressive symptoms. Importantly, a number of studies have shown that serum *BDNF* levels increase with administration of antidepressant medication (39), which is negatively correlated with depressive symptom severity (10, 11). Additionally, Alder and Thakker-Varia (9) suggest that *BDNF* plays a role in neuronal plasticity, particularly emotional processing networks that are compromised in depression. However, further research into the effects of *BDNF* on neuroplasticity and neurodevelopment using post-mortem brain tissue and neuroimaging cohorts with childhood abuse exposure and depressive symptom data are needed before firm conclusions can be made on the moderating mechanisms by which BDNF genotypic variation acts.

### Strengths and Limitations

This study has several notable strengths, including the longitudinal study design, comprehensive covariate adjustment, and centering of data. However, a number of limitations should be acknowledged. First, the assessment of environmental exposures and self-reporting of abuse is limited and subject to bias. Specifically, within the childhood abuse group, only those who had experienced severe childhood abuse either physical or sexual were included in analysis. Emotional abuse or neglect experienced during childhood has also been associated with depressive symptoms (40) yet were not considered in this study. Validity of self-reporting has also been questioned due to significant recall bias, often resulting in under-reporting of abuse (41) in both reporting stressful life events and recalling adverse childhood experiences (41, 42). Thus, our findings may represent a conservative estimate, which would dilute the interaction effect within the sample population and could contribute to negative results. Second, our measures of positive environmental exposure (e.g., positive life events) were sub-optimal and only capture adulthood experiences. Measures of “positive” exposures in childhood, such as maternal attachment or parental engagement, would be ideal but were not available. Third, *BDNF* serum levels were not measured in our study. Given that *BDNF* serum levels have been negatively correlated with depressive symptoms (39) and carriage of the Met allele (13), future study into *BDNF* genetic variation as a moderator of life events on depressive symptoms should include measurement of *BDNF* serum levels. Finally, rather than diagnosis of major depressive disorder, depressive symptom severity was the primary outcome measure limiting extrapolation and comparison to similar studies. However, the PHQ-9 scale consists of the nine diagnostic criteria outlined in DSM-IV and has been validated as a tool for assessing depressive symptom trajectories (20) and may translate better into the primary care setting.

## Conclusion

We have identified a novel three-way interaction between *BDNF* genetic variation, a history of severe childhood abuse, and time that was associated with depressive symptom severity. This interaction highlights the dynamic nature of gene–environment–depression interactions and the importance of longitudinal assessment of these interactions. Discrepancies among previous study results could be associated with not just study design, but also time of analysis. Given this is the first longitudinal study of its kind; replication of results is needed.

## Author Contributions

JG conceived and established the diamond cohort. CB, IE, and JG conceived the current study design and analysis. MP project managed the data collection. CW and CB conducted the analysis and wrote the first draft of the manuscript. All authors contributed to further drafts of the manuscript. All authors have read and approve the current version of the manuscript.

## Conflict of Interest Statement

The authors declare that the research was conducted in the absence of any commercial or financial relationships that could be construed as a potential conflict of interest. The reviewer CO declared a shared affiliation, though no collaboration or input in the work presented in this article, with the authors, to the handling Editor, who ensured that the process nevertheless met the standards of a fair and objective review.
