# Supplementary material for: The Brain-Derived Neurotrophic Factor Val66Met Polymorphism Moderates the Effects of Childhood Abuse on Severity of Depressive Symptoms in a Time-Dependent Manner
Source: Front Psychiatry. 2016 Aug 29;7:151. doi: 10.3389/fpsyt.2016.00151 (PMC5002876; doi:10.3389/fpsyt.2016.00151)
Supplement: Supplementary file 1 [file Table_1.docx]

**SUPPLEMENTARY MATERIAL**

**The brain derived neurotrophic factor (*BDNF*) Val66Met polymorphism moderates the effects of childhood abuse on severity of depressive symptoms in a time dependent manner**

**Table of Contents**

**Supplemental Tables** 2

# Table S1. Life Events Measured 2

**Table S2**. Ancestry Informative Markers by HapMap Population 3

**Table S3**. Raw p values for potential covariates by abuse type, life events, and genotype 4

**Table S4**. Severe child abuse models and backward stepwise penalized likelihood model

selection procedure 5

| \| **Supplementary Table S1.** Life Events Measured \| \| --- \| \| 1. Major personal injury or illness \| \| 2. Major illness or injury of a parent, close family member or friend \| \| 3. The death of a spouse, parent, close family member or friend \| \| 4. Marriage \| \| 5. Separation or divorce due to marital/relationship difficulties or steady relationship breakdown \| \| 6. Hit, slapped, kicked or otherwise physically hurt by partner/ex partner \| \| 7. Hit, slapped, kicked or otherwise physically hurt by partner/ex partner \| \| 8. Partner/ ex partner forced you to have sexual activities \| \| 9. Someone other than partner/ ex partner forced you to have sexual activities \| \| 10. Serious problem with a close friend, neighbour or relative \| \| 11. A major change in financial status \| \| 12. Change in job/workplace (including retirement, being sacked, or made redundant) \| \| 13. Pregnancy or partner's pregnancy \| |
| --- | --- | --- | --- | --- | --- | --- | --- | --- | --- | --- | --- | --- | --- | --- |

| **Supplementary Table S2.** Ancestry Informative Markers by HapMap Population | | |  |
| --- | --- | --- | --- |
| **CEU** | **CHB** | **YRI** | |
| rs1402851 | rs10488619 | rs1368928 | |
| rs16877243 | rs11098964 | rs1446959 | |
| rs1698042 | rs11184898 | rs1494962 | |
| rs2930125 | rs11203006 | rs1563382 | |
| rs2934193 | rs1347201 | rs2388511 | |
| rs3912537 | rs1488299 | rs4241398 | |
| rs4484738 | rs1519260 | rs7158302 | |
| rs4653130 | rs1538956 | rs10933921 | |
| rs4721415 | rs315280 | rs1444893 | |
| rs6510332 | rs36110 | rs1716167 | |
| rs1001484 | rs5753625 | rs1811510 | |
| rs6552216 | rs6595142 | rs1823778 | |
| rs1002587 | rs12595448 | rs1827950 | |
| rs10879311 | rs12644851 | rs1894450 | |
| rs1227647 | rs2416504 | rs2220128 | |
| rs12678324 | rs2486448 | rs2416791 | |
| rs1986420 | rs2927385 | rs2948905 | |
| rs2759281 | rs4240793 | rs4737761 | |
| rs326626 | rs4265409 | rs6785846 | |
| rs6141319 | rs590614 | rs692713 | |

CEU, Northern and Western European in Utah

CHB, Han Chinese in Beijing

YRI, Yoruba in Ibadan, Nigeria

| **Supplementary Table S3.** Raw p values for potential covariates by abuse type, life events, and genotype | | | | | | | | |
| --- | --- | --- | --- | --- | --- | --- | --- | --- |
|  |  |  |  |  | |  | | |
| **Baseline variables (n = 310)** | HoSCA | HoPF | Positive life events | | Negative life events | | BDNF rs6265 |  |
| Age | 0.140 | 0.370 | 0.087 | | **0.016** | | 0.972 |  |
| Sex | 0.078 | **0.002** | 0.524 | | 0.123 | | 0.475 |  |
| English as first language | 0.164 | 0.217 | 0.104 | | 0.414 | | 0.978 |  |
| Northern European genetic ancestry 100% | n/a | n/a | n/a | | n/a | | n/a |  |
| *Depression* |  |  |  | |  | |  |  |
| DSM-IV Major Depressive Disorder, % (n) - 301 | **0.024** | **0.021** | 0.768 | | 0.080 | | 0.177 |  |
| Relative with a history of depression, % (n) - 256 | 0.431 | 0.107 | 0.246 | | 0.750 | | 0.659 |  |
| *Medication Use - last 12m* |  |  |  | |  | |  |  |
| Antidepressant, % (n) | 0.666 | 0.310 | 0.233 | | 0.501 | | 0.631 |  |
| Anxiolytic, % (n) | 0.195 | 0.216 | 0.581 | | 0.227 | | 0.926 |  |
| Sedative, % (n) - 309 | 0.391 | **0.000** | 0.967 | | **0.001** | | 0.095 |  |
| Antipsychotic, % (n) | 0.485 | 0.474 | 0.825 | | **0.046** | | 0.413 |  |
| St John's Wort, % (n) - 309 | 0.346 | 0.073 | 0.502 | | 0.253 | | 0.148 |  |
| *General health* |  |  |  | |  | |  |  |
| Self-rated health, good-to-excellent, % (n) | **0.004** | 0.106 | **0.028** | | 0.295 | | 0.766 |  |
| *Substance use* |  |  |  | |  | |  |  |
| Alcohol abuse/dependence, % (n) - 301 | 0.611 | 0.776 | 0.426 | | 0.905 | | 0.633 |  |
| Substance abuse/dependence (ex. alcohol), % (n) - 301 | **0.009** | 0.434 | 0.312 | | **0.053** | | 0.155 |  |
| Current smoker, % (n) - 309 | 0.055 | 0.220 | 0.511 | | 0.625 | | 0.817 |  |
| *Socioeconomic status* |  |  |  | |  | |  |  |
| Education - 309 | 0.574 | 0.052 | 0.788 | | 0.436 | | 0.433 |  |
| Managing on available income - 308 | 0.189 | **0.019** | 0.149 | | **0.026** | | 0.702 |  |
| *WHO Quality of Life & Functioning* |  |  |  | |  | |  |  |
| Environmental context, mean (sd) | 0.063 | **0.000** | **0.016** | | **0.001** | | 0.872 |  |
| Social context, mean (sd) - 308 | **0.009** | 0.139 | **0.013** | | **0.033** | | 0.705 |  |
| *Severe childhood abuse, % (n)* |  |  |  | |  | |  |  |
| Either physical or sexual or both | x | 0.054 | 0.837 | | 0.795 | | 0.724 |  |
| Both physical and sexual | x | 0.056 | 0.824 | | 0.294 | | 0.557 |  |
| Only physical | x | 0.237 | 0.516 | | 0.733 | | 0.273 |  |
| Only sexual | x | **0.040** | 0.901 | | 0.733 | | 0.991 |  |
| *Partner abuse, % (n)* |  |  |  | |  | |  |  |
| Ever afraid of partner, % (n) | 0.054 | x | 0.223 | | **0.017** | | 0.909 |  |
| *Visited counsellor/psychologist/psychiatrist past 12m, % (n)* |  |  |  | |  | |  |  |
| One or more treatment sessions, % (n) | 0.277 | **0.002** | 0.076 | | 0.160 | | 0.298 |  |
| *Life events in past 12 months, % (n)* |  |  |  | |  | |  |  |
| At least one positive | 0.837 | 0.223 | x | | **0.027** | | 0.396 |  |
| At least one negative | 0.795 | **0.017** | **0.027** | | x | | 0.938 |  |
| **Bolded values p< 0.05**. HoSCA – History of severe child abuse; HoPF – History of partner fear; WHO – The World Health Organization; Psychosocial – at least one visit to counsellor/psychologist/psychiatrist past 12months | | | | | | | | |

| **Supplementary Table S4.** Severe child abuse models and backward stepwise penalized likelihood model selection procedure | | | | | |  |
| --- | --- | --- | --- | --- | --- | --- |
|  |  |  | **Backward stepwise models** | |  |  |
| **Model terms** | **Unadjusted Model** | **Full adjusted model** | **Final Adjusted Model** |  |  |  |
| Intercept | x | x | x |  |  |  |
| Time | x | x | x |  |  |  |
| Genotype – rs6265 | x | x | x |  |  |  |
| Severe child abuse | x | x | x |  |  |  |
| rs6265 x Time | x | x | x |  |  |  |
| Severe child abuse x Time | x | x | x |  |  |  |
| rs6265 x Severe child abuse | x | x | x |  |  |  |
| rs6265 x Severe child abuse x Time | x | x | x |  |  |  |
| DSM IV Depression diagnosis |  | x | x |  |  |  |
| DSM IV Depression diagnosis x Time |  | x | x |  |  |  |
| DSM IV Depression diagnosis x rs6265 |  | x | x |  |  |  |
| DSM IV Depression diagnosis x Severe child abuse |  | x | x |  |  |  |
| Self Rated Health |  | x | x |  |  |  |
| Self Rated Health x Time |  | x | x |  |  |  |
| Self Rated Health x rs6265 |  | x | x |  |  |  |
| Self Rated Health x Severe child abuse |  | x | x |  |  |  |
| DSM IV substance abuse/addiction |  | x | x |  |  |  |
| DSM IV substance abuse/addiction x Time |  | x | x |  |  |  |
| DSM IV substance abuse/addiction x rs6265 |  | x | x |  |  |  |
| DSM IV substance abuse/addiction x Severe child abuse |  | x | x |  |  |  |
| WHOQOL Social |  | x | x |  |  |  |
| WHOQOL Social x Time |  | x |  |  |  |  |
| WHOQOL Social x rs6265 |  | x | x |  |  |  |
| WHOQOL Social x Severe child abuse |  | x | x |  |  |  |
|  |  |  |  |  |  |  |
| ***Bayesian Information Criterion (BIC)*** | 10392.723 | 10051.694 | 10044.263 |  |  |  |
| ***Inter-model BIC difference*** |  |  | -7.431 |  |  |  |
|  |  |  |  |  |  | |
